# Construction of Lentiviral Vectors Carrying Six Pluripotency Genes in Yak to Obtain Yak iPSC Cells

**DOI:** 10.3390/ijms25179431

**Published:** 2024-08-30

**Authors:** Ruilin Zeng, Xianpeng Huang, Wei Fu, Wenhui Ji, Wenyi Cai, Meng Xu, Daoliang Lan

**Affiliations:** 1 College of Animal & Verterinary Sciences, Southwest Minzu University, Chengdu 610041, China; 2 Key Laboratory of Qinghai-Tibet Plateau Animal Genetic Resource and Utilization, Ministry of Education, Southwest Minzu University, Chengdu 610041, China

**Keywords:** yak, lentivirus, pluripotency gene, reprogramming

## Abstract

Yak is an excellent germplasm resource on the Tibetan Plateau and is able to live in high-altitude areas with hypoxic, cold, and harsh environments. Studies on induced pluripotent stem cells (iPSCs) in large ruminants commonly involve a combination strategy involving six transcription factors, *Oct4*, *Sox2*, *Klf4*, *c-Myc*, *Nanog*, and *Lin28* (OSKMNL). This strategy tends to utilize genes from the same species to optimize pluripotency maintenance. In this study, we cloned the six pluripotency genes (OSKMNL) from yak and constructed a multi-cistronic lentiviral vector carrying these genes. This vector efficiently delivered the genes into yak fibroblasts, aiming to promote the reprogramming process. We verified that the treated cells had several pluripotency characteristics, marking the first successful construction of a lentiviral system carrying yak pluripotency genes. This achievement lays the foundation for subsequent establishment of yak iPSCs and holds significant implications for yak-breed improvement and germplasm-resource conservation.

## 1. Introduction

Embryonic stem cells (ESCs) originate from the inner cell mass of early mammalian embryos. They possess unlimited proliferation capacity and full lineage-differentiation potential [1]. However, the acquisition and application of ESCs involve ethical concerns, necessitating alternative methods to obtain cells with similar functions. Takahashi [2] and Yu [3] successfully induced mouse and human fibroblasts to generate induced pluripotent stem cells (iPSCs) by introducing transcription factors such as *Oct4*, *Sox2*, *Klf4*, *c-Myc*, *Nanog*, and *Lin28*. These iPSCs possess the ability for unlimited self-renewal and proliferation. They can also differentiate into all cell types of the three germ layers [2,3]. Somatic cells from animals such as pigs [4,5], dogs [6], goats [7,8], and cattle [9,10,11] have also been successfully reprogrammed into iPSCs. Notably, when inducing iPSCs from large domestic animals especially ruminants, additional transcription factors such as Nanog and Lin28 are generally added alongside the four OSKM factors [10,12,13,14]. Furthermore, genes from the original animals are used to optimize reprogramming efficiency and enhance the maintenance of pluripotency [5,15,16].

Yaks (*Bos grunniens*) are uniquely adapted to live in the high-altitude regions of the Qinghai–Tibet Plateau, characterized by hypoxic, cold, and harsh environments. They represent a valuable livestock resource native to this area. Yaks are also a primary economic resource for pastoralists in cold regions of China, providing essential supplies such as meat, milk, and hides [17]. However, under natural grazing conditions, yak populations face issues such as imbalanced herd structures, loss of superior genetic resources, and decline in biological performance, including reduced fertility, extended growth cycles, weight loss, and weakened resistance [18]. Given the significant potential of iPSC technology in genetic-resource preservation, transgenic breeding, and livestock-industry innovation [19], its application in the yak field can positively impact population improvement, genetic-diversity conservation, and the sustainable development of animal husbandry.

Lentiviral vectors (LV) have become effective and versatile tools for gene delivery into dividing and non-dividing cells, in vivo and in vitro [20]. In the present study, we innovatively constructed two multi-cistronic LV systems, FUW-tetO-OSM-EGFP and FUW-tetO-KNL-mCherry, by cloning six key transcription factors from yak, namely, *Oct4*, *Sox2*, *Klf4*, *c-Myc*, *Nanog*, and *Lin28*. These vectors aimed to achieve efficient and controllable expression of transcription factors. By transfecting yak fibroblasts with these vectors, we provided a feasible method for the subsequent establishment of stable yak iPSCs cell lines. Our results also lay the foundation for the efficient utilization and improvement of yak genetic resources.

## 2. Results

### 2.1. Cloning of Six Pluripotency-Related Transcription Factors in Yak

**Figure 1:**
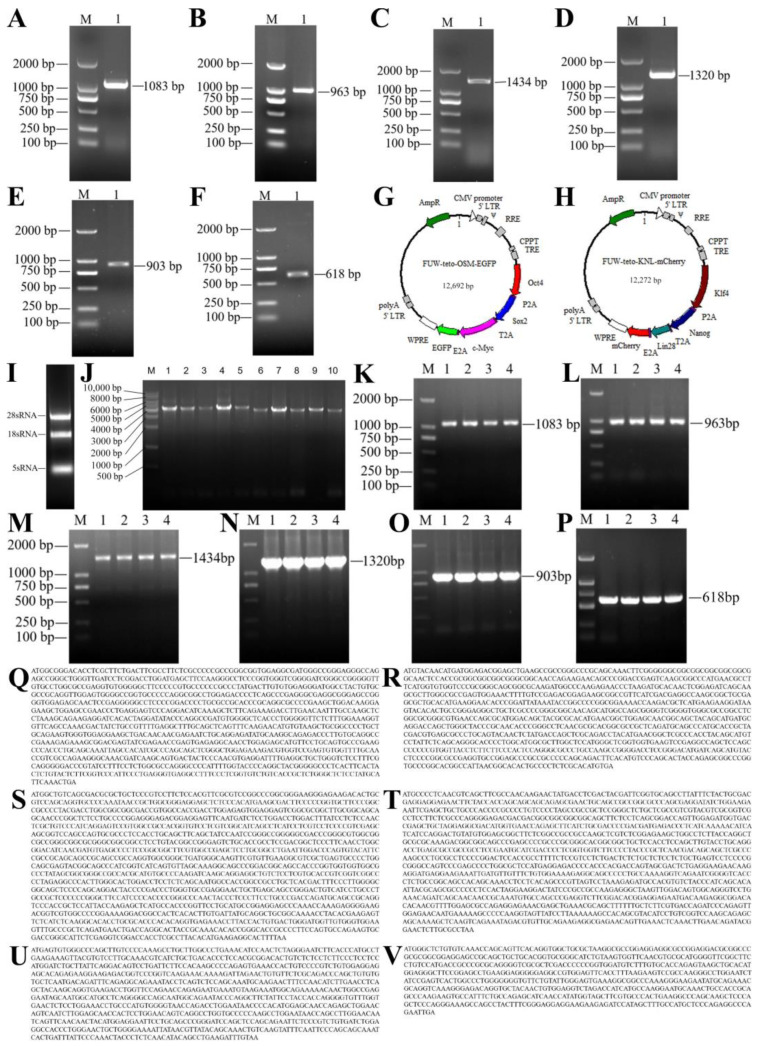
Construction of lentiviral vectors FUW-tetO-OSM-EGFP and FUW-tetO-KNL-mCherry. (**A**–**F**) PCR amplification results of *Oct4*, *Sox2*, *Klf4*, *c-Myc*, *Nanog*, and *Lin28* genes; M represents marker 2000, 1 represents PCR product; (**G**) Structure of FUW-tetO-OSM-EGFP lentiviral vector; (**H**) Structure of FUW-tetO-KNL-mCherry lentiviral vector; (**I**) Electrophoresis of total RNA from yak gonadal ridge; (**J**) PCR amplification results of lentiviral vectors; M represents marker 2000, 1–5 represent FUW-tetO-OSM-EGFP plasmid PCR products (4317 bp), 6–10 represent FUW-tetO-KNL-mCherry plasmid PCR products (3897 bp); (**K**–**P**) PCR amplification results of *Oct4*, *Sox2*, *Klf4*, *c-Myc*, *Nanog*, and *Lin28* genes from plasmid; M represents marker 2000, 1–4 represent plasmid PCR products; (**Q**–**V**) Sequence information of *Oct4*, *Sox2*, *Klf4*, *c-Myc*, *Nanog*, and *Lin28* genes of yak.

Electrophoretic analysis of total RNA extracted from yak fetal genital ridges showed that the RNA samples had good integrity, presenting clear and complete band structures (Figure 1I). Using this RNA as a template, cDNA was synthesized by reverse transcription. The specific amplification of key pluripotency genes in yak, including *Oct4*, *Sox2*, *Klf4*, *c-Myc*, *Nanog*, and *Lin28*, was performed using RT-PCR. The amplification products were separated by 1% agarose-gel electrophoresis, confirming the expected gene-coding region lengths as follows: *Oct4* at 1083 bp, *Sox2* at 963 bp, *Klf4* at 1434 bp, *c-Myc* at 1320 bp, *Nanog* at 903 bp, and *Lin28* at 618 bp (Figure 1A–F,Q–V). The amplified yak OSKMNL gene fragments were subsequently cloned into the pMD19-T vector, successfully constructing six recombinant plasmids: pMD19T-Oct4, pMD19T-Sox2, pMD19T-Klf4, pMD19T-c-Myc, pMD19T-Nanog, and pMD19T-Lin28. These recombinant plasmids were transformed into DH5α *E. coli* competent cells and identified by colony PCR. All plasmid transformants showed PCR amplification bands of the expected sizes (Figure 1K–P), further confirming the correctness and stability of the plasmid constructions.

### 2.2. Construction of the Lentiviral Vectors FUW-tetO-OSM-EGFP and FUW-tetO-KNL-mCherry

Based on the FUW vector backbone, we successfully constructed two LVs carrying different combinations of pluripotency genes. The first vector, FUW-tetO-OSM-EGFP, integrated the yak *Oct4*, *Sox2*, and *c-Myc* genes and simultaneously expressed the green fluorescent protein EGFP (Figure 1G). The second vector, FUW-tetO-KNL-mCherry, incorporated the *Klf4*, *Nanog*, and *Lin28* genes and carried the red fluorescent protein mCherry gene (Figure 1H). This design aimed to distinguish and track the expression of these two key gene sets through different fluorescent markers. The constructed plasmids were transformed into competent cells, and five single colonies for each plasmid were picked for PCR verification. Results showed that all picked colonies produced single and bright bands, strongly confirming the accuracy of the vector construction and the consistency of the recombinant plasmids (Figure 1J).

### 2.3. Immunofluorescence of Lentiviral Packaging and Transfection of 293T Cells

**Figure 2:**
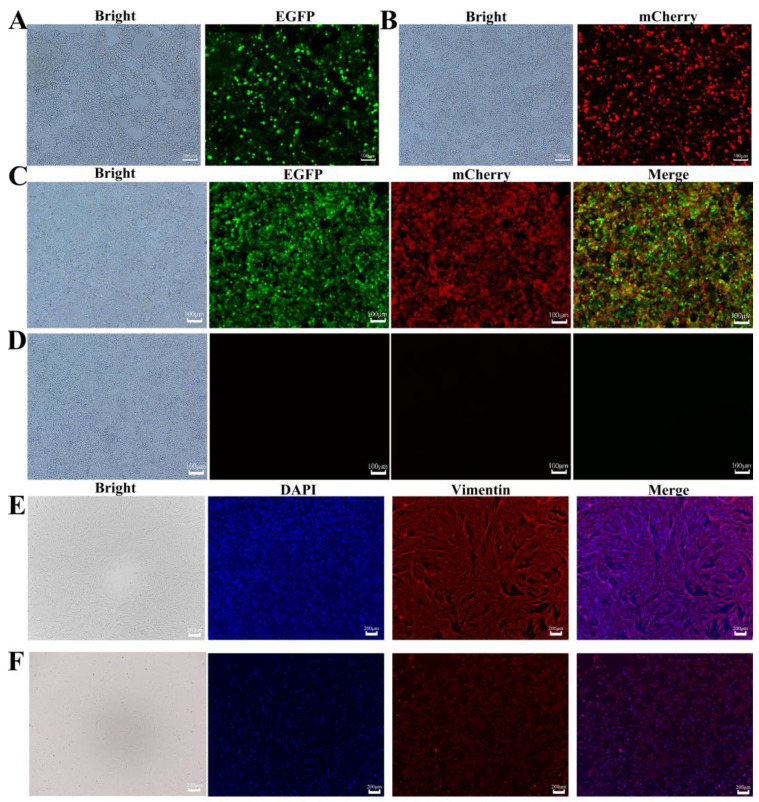
Lentiviral transduction of 293T cells and immunofluorescence identification of fibroblasts. (**A**) Packaging results of FUW-tetO-OSM-EGFP lentiviral vector; (**B**) Packaging results of FUW-tetO-KNL-mCherry lentiviral vector; (**C**,**D**) Expression of fluorescent proteins in 293T cells after lentiviral transduction; (**C**) is the experimental group, (**D**) is the control group; (**E**) Immunofluorescence identification of yak fibroblasts; (**F**) Immunofluorescence identification of mouse fibroblasts, with red fluorescence indicating vimentin.

Using Lipofectamine 2000 transfection reagent, we co-transfected 293T cells with the constructed FUW-tetO-OSM-EGFP and FUW-tetO-KNL-mCherry plasmids along with packaging plasmids. After 72 h, green and red fluorescence was observed under a fluorescence microscope (Figure 2A,B), demonstrating successful plasmid transfection and normal gene expression.

Furthermore, 293T cells were transfected with the lentiviral particles produced from the above transfection. Similarly, after 72 h, cells in the transfected groups exhibited distinct green and red fluorescence under a fluorescence microscope (Figure 2C), whereas the blank control group showed no fluorescence (Figure 2D). This result clearly confirmed that the packaged lentiviruses were successfully produced and exhibited high infection efficiency and expression capability.

### 2.4. Identification of Yak and Mouse Embryonic Fibroblasts (MEFs)

Using an inverted microscope, we observed yak fibroblasts (Figure 2E) and mouse fibroblasts (Figure 2F). These cells primarily exhibited spindle-shaped and irregular triangular forms with distinct edges, displaying typical reticular or radial growth patterns characteristic of fibroblasts. To further confirm cell type and purity, we used vimentin as a specific marker and performed immunofluorescence staining on third-passage fibroblasts. Under a fluorescence microscope, red fluorescence distinctly marked vimentin expression, whereas blue fluorescence indicated the positions of cell nuclei (Figure 2E,F). Notably, the majority of cells showed strong red fluorescence signals, which robustly confirmed that the cultured cell populations were fibroblasts with very high purity.

### 2.5. Reprogramming of Yak Fibroblasts

**Figure 3:**
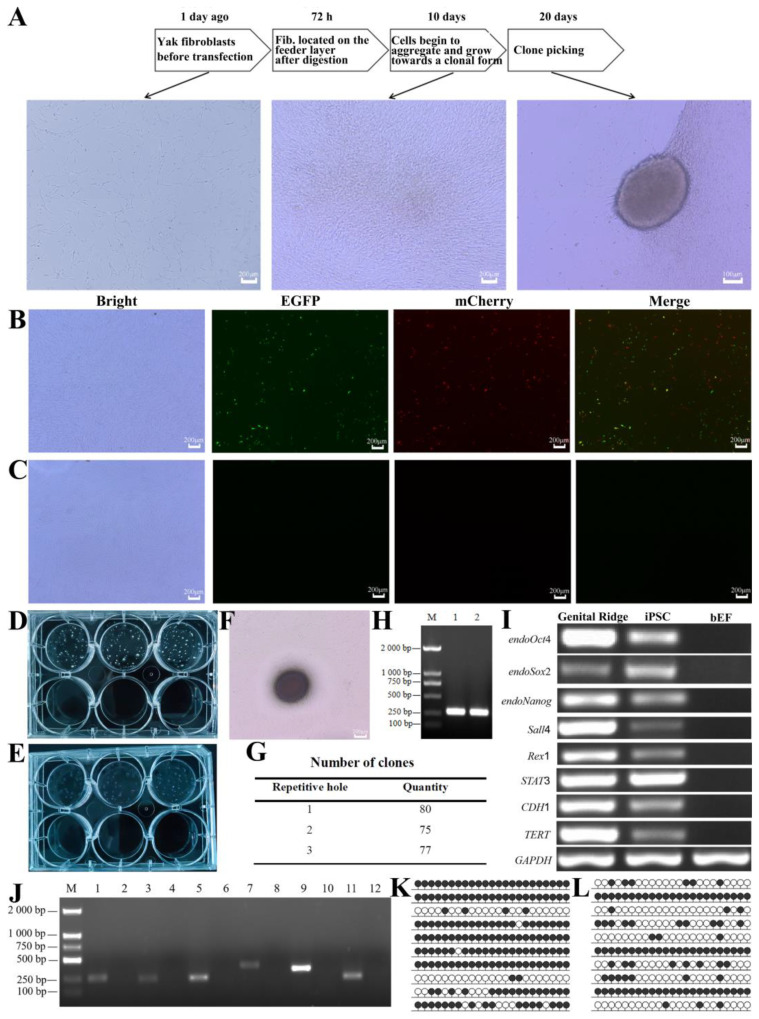
Reprogramming of yak fibroblasts and preliminary pluripotency identification. (**A**) Reprogramming steps and morphological changes of yak fibroblasts; (**B**,**C**) Expression of fluorescent proteins in yak fibroblasts after lentiviral transduction; (**B**) is the experimental group, (**C**) is the control group; (**D**–**G**) AP staining of yak iPSCs; (**D**) shows the clone morphology on a six-well plate before staining, (**E**) shows the clone morphology on a six-well plate after staining, (**F**) shows AP-stained clones, (**G**) shows the number of positive clones in three replicate wells; (**H**) PCR for the methylation-status detection of the *Oct4* promoter region; (**I**) RT-PCR detection of pluripotency-related genes in yak iPSCs, with gonadal ridge as the positive control and BEF as the negative control; (**J**) RT-PCR detection of related genes in fibroblasts after lentiviral transduction; M is marker 2000. Experimental group: 1 for *Oct4* (242 bp), 3 for *Sox2* (215 bp), 5 for *c-Myc* (221 bp), 7 for *Klf4* (354 bp), 9 for *Nanog* (309 bp), 11 for *Lin28* (204 bp); control group: 2 for *Oct4*, 4 for *Sox2*, 6 for *c-Myc*, 8 for *Klf4*, 10 for *Nanog*, 12 for *Lin28*; (**K**) Methylation status of the *Oct4* promoter region in yak fibroblasts; (**L**) Methylation status of the *Oct4* promoter region in yak iPSCs.

The reprogramming experiment followed the predetermined protocol (Figure 3A). Yak fibroblasts were observed under an inverted fluorescence microscope 72 h after lentiviral transduction. The experimental group showed green and red fluorescence signals (Figure 3B), whereas the blank control group showed no fluorescence (Figure 3C). RT-PCR results further indicated that the experimental group successfully amplified DNA bands of the expected size, whereas no bands were observed in the control group (Figure 3J). These results demonstrated that the constructed LVs successfully transduced yak fibroblasts and effectively expressed the genes within the cells.

### 2.6. AP Staining, Pluripotency Gene-Expression Analysis, and Epigenetic Characterization of Yak iPSCs

Successfully transfected cells were seeded onto MEF feeder layers and transferred into a specialized iPSCs culture medium for subsequent cultivation. During the reprogramming process, the cell morphology underwent significant changes (Figure 3A). Initially, spindle-shaped and irregular cells gradually transformed into round shapes and began to grow in clusters. By approximately day 10, the clustering became more pronounced. Subsequently, these clusters started to change into colony-like structures, eventually forming distinct, independent colonies clearly separated from the feeder layer. After day 20, these colonies were able to be selected for further expansion and pluripotency assessment.

Alkaline phosphatase (AP) staining of yak iPSCs showed a purple-black positive reaction, whereas the feeder layer cells remained unstained, indicating a negative reaction (Figure 3D–F). Counting the number of positive colonies in three replicate wells yielded an average of 77.33 ± 2.52 colonies (Figure 3G), further confirming the success of the reprogramming process. To more comprehensively assess pluripotency, we used RT-PCR to detect the expression levels of pluripotency-related genes, including *Oct4*, *Sox2*, *Nanog*, *Sall4*, *Rex1*, *STAT3*, *CDH1*, and *TERT*, in yak iPSCs. Gonadal ridge tissue served as a positive control, whereas BEF served as a negative control. Results showed that yak iPSCs significantly expressed these key pluripotency genes (Figure 3I).

To investigate epigenetic changes, we utilized bisulfite sequencing to examine the methylation status of the CpG island in the promoter region of the *Oct4* gene in yak iPSCs and fibroblasts by using fibroblasts as a baseline comparison. The process included the bisulfite treatment of genomic DNA to specifically recognize and enrich methylated CpG sites, followed by PCR amplification of the *Oct4* promoter region by using designed primers (Figure 3H). The amplification products were transformed into bacteria, and single colonies were picked for sequencing. Sequencing results revealed that the *Oct4* gene exhibited relatively high methylation levels in fibroblasts (Figure 3K), whereas these levels were significantly reduced in yak iPSCs (Figure 3L). This difference indicated significant epigenetic remodeling during reprogramming.

## 3. Discussion

Pluripotent stem cells show great potential in livestock genetic improvement and germplasm-resource conservation. Establishing stem-cell lines in large livestock remains challenging, but it has been enabled by the emergence of iPSCs technology [13]. Previous successful applications of iPSCs in animals such as pigs, including the generation of chimeric pigs [21] and cloned pigs [22], provide favorable evidence for using iPSCs in yaks and other livestock for breed improvement and genetic-resource conservation.

*Oct4*, *Sox2*, *Klf4*, *c-Myc*, *Nanog*, and *Lin28* are universally present in pluripotent stem cells and are crucial to maintaining stem-cell characteristics and promoting somatic-cell reprogramming [23,24]. Among them, *Oct4*, *Sox2*, and *Nanog* serve as core marker genes that cooperatively regulate hundreds of target sites, thereby maintaining the pluripotency transcriptional regulatory network [25,26]. Although the oncogenic potential of *Klf4* and *c-Myc* raises concerns, they are indispensable for enhancing reprogramming efficiency and balancing cell proliferation and apoptosis through complex mechanisms [22,27,28]. Specifically, *c-Myc* can promote p53-mediated apoptosis, whereas *Klf4* mitigates this effect by inhibiting p53 expression and regulating the cell cycle through P21 activation [29,30]. Additionally, *c-Myc* promotes chromatin opening and co-activates pluripotency genes with *Oct4* and *Sox2*. Meanwhile, *Klf4* upregulates *Nanog* via the STAT3 pathway while inhibiting endoderm differentiation genes [31]. *Lin28* regulates mRNA stability by influencing the maturation of let-7 miRNA, thereby promoting self-renewal and significantly enhancing reprogramming efficiency [3,32]. Therefore, the intricate interactions and balanced regulatory mechanisms among these factors are critical for the efficient reprogramming of somatic cells to a pluripotent state.

Takahashi [2] and Yu [3] identified *Oct4*, *Sox2*, *Klf4*, *c-Myc*, *Nanog*, and *Lin28* from numerous transcription factors associated with stem-cell pluripotency. They demonstrated that two different combinations of these factors, *Oct4*, *Sox2*, *Klf4*, *c-Myc* (OSKM) and *Oct4*, *Sox2*, *Nanog*, *Lin28* (OSNL), can reprogram somatic cells into pluripotent stem cells. However, the ectopic expression of OSKM or OSNL alone was not sufficient for stable induction of pluripotency in bovine fibroblasts. The combination of all six factors (OSKMNL) enhances the efficiency and stability of iPSCs induction in humans, mice, and other species [33,34]. Notably, the ectopic expression of *NANOG* is necessary for the generation and maintenance of biPSCs from bovine fibroblasts [35]. This combination facilitates the reprogramming of somatic cells in large ruminant livestock, such as pig [21,36,37], cattle [35,38], and sheep [39], which are typically more challenging to reprogram.

In this study, we cloned six genes from yak: *Oct4*, *Sox2*, *Klf4*, *c-Myc*, *Nanog*, and *Lin28*. We innovatively constructed two multi-cistronic LV systems: the FUW-tetO-OSM-EGFP vector, integrating the *Oct4*, *Sox2*, and *c-Myc* genes, and the FUW-tetO-KNL-mCherry vector, containing the *Klf4*, *Nanog*, and *Lin28* genes. These vectors were then transfected into yak fibroblasts. After AP staining, RT-PCR detection of pluripotency-related gene expression, and methylation-level analysis, we found that the treated cells exhibited a series of characteristics similar to known iPSCs from other species. These characteristics included enhanced AP activity, significant expression of the pluripotency genes *Oct4*, *Sox2*, and *Nanog*, and associated DNA demethylation. All are important markers of the acquisition of a pluripotent state.

In conclusion, our research successfully constructed the first multi-cistronic LV system carrying six yak pluripotency genes. We also found that these vectors may induce the reprogramming of yak fibroblasts. This study lays the foundation for establishing yak iPSC lines induced by specific yak transcription factors and further exploring yak iPSCs. It has significant implications for yak-breed improvement and germplasm-resource conservation.

## 4. Materials and Methods

### 4.1. Cloning of Six Pluripotency-Associated Transcription Factors in Yak

Genital ridge tissues, appearing as white rice-grain-like structures, were collected from both sides of the abdominal wall of 3- to 5-month-old yak fetuses. Total RNA was extracted using Trizol (Takara, Osaka, Japan). Based on the known gene sequences of bovine *Oct4*, *Sox2*, *Klf4*, *c-Myc*, *Nanog*, and *Lin28* from the National Center for Biotechnology Information (NCBI) database, cloning primers were designed (Table 1) to amplify the corresponding six transcription factor genes in yak. The amplified products were purified and recovered using a gel-extraction kit (Omega, Norcross, GA, USA). The recovered DNA fragments were then ligated into the pMD19-T vector to construct recombinant plasmids. Colonies suspected to contain positive inserts were selected for expansion. To verify the successful construction of the recombinant vectors and the accuracy of sequences, secondary PCR amplification and sequencing analysis were performed on these cultured colonies.

### 4.2. Construction of Lentiviral Vectors

Referring to the previously cloned sequences of yak *Oct4*, *Sox2*, *Klf4*, *c-Myc*, *Nanog*, and *Lin28* genes, as well as EGFP, mCherry, and 2A peptide sequences, seamless cloning primers were designed (Table 2). Using these primers, *Oct4*, *Sox2*, *Klf4*, *c-Myc*, *Nanog*, *Lin28*, *EGFP*, and *mCherry* target-gene fragments were amplified from pMD19T-Oct4, pMD19T-Sox2, pMD19T-Klf4, pMD19T-c-Myc, pMD19T-Nanog, pMD19T-Lin28, FUW-tetO-EGFP, and pCMV-mCherry-MCS-Neo plasmids as templates. After gel extraction, the amplified products were stored at −20 °C. Next, the FUW-tetO-MCS plasmid was double-digested with BamH I and Xba I, followed by gel extraction. According to the instructions of the seamless cloning kit, the digested FUW-tetO-MCS was ligated with *Oct4*, *Sox2*, *c-Myc*, and EGFP at a molar ratio of 1:1:1:1 to construct the FUW-tetO-OSM-EGFP LV. Similarly, FUW-tetO-MCS was ligated with *Klf4*, *Nanog*, *Lin28*, and mCherry to construct the FUW-tetO-KNL-mCherry LV. After constructing the vectors, the ligation products were transformed into DH5α competent cells. Positive colonies were selected for expansion, followed by secondary PCR amplification and sequencing verification.

### 4.3. Lentiviral Packaging and Transfection of 293T Cells

We thawed and passaged 293T cells once, and then replaced the culture medium with serum-reduced medium Opti-MEM (Gibco, Shanghai, China) before transfection. According to the Lipo2000 (Invitrogen, Shanghai, China) manual, the constructed LV, the auxiliary packaging plasmid PSPAX2, and the envelope plasmid PMD2.G (TransGen Biotech, Chengdu, China) were co-transfected into 293T cells. Six hours after transfection, we replaced the medium with Dulbecco’s Modified Eagle Medium (DMEM; Gibco) containing 10% FBS (BI, HaEmek, Israel) and 1% Penicillin-Streptomycin Solution (Gibco). The cell supernatant was collected at 48 and 72 h post-transfection, passed through a 0.45 μm filter, and concentrated using a viral concentration kit. The concentrated viral solution was stored at −80 °C.

The day before transfection, healthy 293T cells were seeded onto six-well plates. Subsequently, FUW-tetO-OSM-EGFP and FUW-tetO-KNL-mCherry LVs were transfected into the 293T cells, with the FUW-M2rtTA LV serving as the control group. Polybrene at a concentration of 8 μg/mL was added during transfection to enhance efficiency. After 24 h, the medium was replaced with fresh DMEM complete culture medium. After 72 h of culture, the fluorescence expression of 293T cells was observed using a fluorescence microscope (ZEISS, LSM900, Oberkochen, Germany).

### 4.4. Isolation and Immunofluorescence Identification of Yak Fibroblasts

Three- to five-month-old yak fetuses were selected by strict disinfection procedures, rapid extraction of fetuses, and isolation of skin tissue. The tissue samples were immediately placed in sterile PBS buffer. Under sterile conditions, the tissues were minced into small pieces and digested with a combination of trypsin (Gibco) and collagenase (Worthington, Freehold, NJ, USA). Gentle mechanical trituration and filtration through an 80 μm mesh were performed to remove residual-tissue debris. The harvested cell suspension was centrifuged and then resuspended in culture flasks containing DMEM complete medium. The cells were cultured in an incubator at 37 °C with 5% CO_2_ and appropriate humidity. Once the cells adhered and grew to near confluence, the first medium change and passage were performed, and yak fetal fibroblasts were successfully obtained.

Fibroblasts are typically identified through morphological observation and immunofluorescence staining. First, yak fibroblasts were fixed at room temperature by using 4% paraformaldehyde. Second, vimentin (Boster, Rabbit, 1:100) was used as the primary antibody for incubation, and goat anti-rabbit IgG coupled with Alexa Fluor™ 594 (Invitrogen, 2 μg/mL) was used as the secondary antibody. Observations were made under a fluorescence microscope.

### 4.5. Isolation of MEF and Preparation of Feeder Layers

ICR pregnant mice at E 12.5–13.5 were selected, and the uteri containing embryos were removed and placed in sterile PBS buffer. The mouse embryos were then isolated from the uteri. Normal embryos were selected, and the head, tail, limbs, and abdominal viscera were removed, retaining only the trunk portion. The remaining steps were the same as those for the isolation of yak fetal fibroblasts.

For the third-to-fifth passage MEFs cultured to 90% confluence, we followed these steps for mitomycin-C (MMC, Millipore, Billerica, MA, USA) treatment and cryopreservation. First, the original culture medium was discarded, and the cells were washed twice with PBS. Next, culture medium containing 10 μg/mL MMC was added to the cells, and they were incubated at 37 °C in a 5% CO_2_ incubator for 3 h. After treatment, the MMC-containing medium was carefully discarded, and the cells were washed five times with PBS to ensure complete removal of MMC. The cells were then trypsinized and cryopreserved (90% FBS + 10% DMSO) for future use.

### 4.6. Lentiviral Transfection of Yak Fibroblasts

Healthy yak fibroblasts were digested with trypsin and seeded onto six-well plates at a density of 5 × 10^4^ cells per well. Before transfection, the cells were washed twice with PBS, and 2 mL of medium containing 8 μg/mL Polybrene was added. The three lentiviruses FUW-teto-OSM-EGFP, FUW-teto-KNL-mCherry, and FUW-M2rtTA were co-transfected into the yak fibroblasts. After 24 h of transfection, the medium was discarded and replaced with DMEM complete medium containing 1 μg/mL Dox for continued culture. After 72 h of transfection, the cells were digested with trypsin and seeded onto six-well plates containing feeder-layer MEFs at a density of 1 × 10^4^ cells per well. The culture medium was replaced with medium optimized for iPSCs. On days 20–30, cell colonies were mechanically isolated and reseeded onto the feeder layer.

The feeder layer was prepared as follows: six-well plates were coated with 0.1% gelatin for 30 min, and the gelatin was discarded for later use. The prepared feeder layer cells were thawed in a 37 °C water bath and seeded onto six-well plates at a density of 5 × 10^4^ cells/cm^2^. The optimized iPSC culture medium included 15% FBS, 1% penicillin–streptomycin, 1% GlutaMAX (Gibco), 1% NEAA (Gibco), 0.1% β-mercaptoethanol (Gibco), 1000 IU/mL LIF (Sigma, Darmstadt, Germany), 4 ng/mL FGF-2 (PeproTech, Cranbury, NJ, USA), 1 μg/mL Dox, and high-glucose DMEM. The culture conditions were 37 °C, 5% CO_2_, and saturated humidity.

### 4.7. Passage Culture of Yak iPSCs

The day before passaging, feeder layer cells were seeded onto 96-well plates and incubated at 37 °C with 5% CO_2_ for subsequent use. Using an inverted microscope, the six-well plates containing yak iPSCs were carefully examined to identify and select target colonies. A 100 μL pipette tip was used to gently detach the selected colonies from the dish bottom. The colonies were then transferred onto another 96-well plate without feeder layers and dispersed by gentle pipetting. Finally, the dispersed colony fragments were seeded onto the precoated feeder layer on the 96-well plates and further cultured to promote their growth. With increased colony size, the cells were gradually passaged onto 24-well plates and subsequently onto six-well plates for continued culture.

With increased number of colonies, TrypLE™ Express (Gibco) was used for cell passaging. The specific procedure was as follows. First, the original culture medium was aspirated, and the cells were washed twice with PBS. Second, 1 mL of TrypLE™ Express was added. When the cells were observed to detach from the dish bottom under a microscope, an excess of PBS was immediately added to terminate the digestion. The cells were then transferred into a 15 mL centrifuge tube and thoroughly dispersed by repeated pipetting. Subsequently, the cells were centrifuged at 1500 rpm for 5 min, and the supernatant was discarded.

An appropriate amount of iPSC culture medium was added to resuspend the cells, which were then seeded onto 35 mm culture dishes and incubated for 30 min. This step aimed to promote the adherence of any feeder layer cells that may be mixed in. Finally, the cell suspension was transferred at a 1:2 ratio onto six-well plates precoated with feeder layers and continued to be cultured to maintain and expand the iPSC cell population.

### 4.8. Alkaline Phosphatase Staining of Yak iPSCs

Staining was performed according to the instructions of an alkaline phosphatase (AP) detection kit (Beyotime, Shanghai, China). First, the iPSC culture medium was aspirated, and the cells were washed three times with PBS. Then, 4% paraformaldehyde was added for fixation for 20 min. The cells were washed three more times with PBS, and then a BCIP/NBT staining working solution was added. The cells were incubated in darkness at room temperature for 1–2 h. The reaction was terminated by washing the cells twice with PBS. Finally, the cells were observed and photographed under a microscope.

### 4.9. RT-PCR Detection of Pluripotency Genes in Yak iPSCs

Total RNA was extracted using Trizol, and cDNA was synthesized according to the instructions of the reverse-transcription kit (Takara). The cDNA was then used as a template for PCR amplification with specific primers for each gene (primer information is provided in Table 3). The amplification products were detected by 1% agarose-gel electrophoresis.

### 4.10. Bisulfite Sequencing of Oct4 Gene

First, high-purity genomic DNA was extracted from yak iPSCs and fibroblasts by using a genomic DNA extraction kit (Tiangen, Beijing, China) according to the manufacturer’s instructions. The genomic DNA was then chemically treated using a DNA bisulfite conversion kit (Beyotime). Using the converted DNA as a template, PCR amplification was performed with a pair of specifically designed primers: the forward primer sequence was GATTTGGATGAGTTTTTAAGGGTT, and the reverse primer sequence was ACTCCAACTTCTCCTTATCCAACTT. The amplification products were then ligated into the pMD19-T vector to construct recombinant plasmids. After bacterial transformation, single-colony selection, and colony PCR verification, at least 10 positive clones were selected for sequencing analysis.

## Figures and Tables

**Table 1:** Primer information of genes.

Gene	Primer (5′-3′)	Product Length (bp)
*Oct4*	F: ATGGCGGGACACCTCGCTT	1083
	R: TCAGTTTGAATGCATAGGAGAGCC	
*Sox2*	F: ATGTACAACATGATGGAGACGG	963
	R: TCACATGTGCGAGAGGGG	
*Klf4*	F: ATGGCTGTCAGCGACGC	1434
	R: TTAAAAGTGCCTCTTCATGTGTAAG	
*c-Myc*	F: ATGCCCCTCAACGTCAGCT	1320
	R: TTAGGCGCAAGAGTTCCGTAT	
*Nanog*	F: ATGAGTGTGGGCCCAGCTTGT	903
	R: TTACAAATCTTCAGGCTGTATGTT	
*Lin28*	F: ATGGGCTCTGTGTCAAACCAG	618
	R: TCAATTCTGGGCCTCTGGGAG	

F: Forward primer; R: Reverse primer.

**Table 2:** Primer information of expression vectors.

Gene	Primer (5′-3′)	Product Length (bp)
*Oct4*	F: AATTACAGGCTAGCTATCAGGCCACCATGGCGGGACACCTCGCTT	1148
	R: ACCTGCTTGCTTTAGCAGAGAGAAGTTTGTGGCGCCGCTGCCGTTTGAATGCATAGGAGAGCCCAG	
*Sox2*	F: CTGCTAAAGCAAGCAGGTGATGTTGAAGAAAACCCCGGGCCTATGTACAACATGATGGAGACGGAG	1043
	R: TCCCCGCATGTTAGAAGACTTCCCCTGCCCTCGCCGGAGCCCATGTGCGAGAGGGGCAG	
*c-Myc*	F: TCTTCTAACATGCGGGGACGTGGAGGAAAATCCCGGCCCAATGCCCCTCAACGTCAGC	1404
	R: TCTCCAGCCAATTTCAAGAGAGCATAATTAGTACACTGGCCCGAGCCGGCGCAAGAGTTCCGTAT	
*EGFP*	F: CTTGAAATTGGCTGGAGATGTTGAGAGCAACCCAGGTCCCATGGTGAGCAAGGGCGAG	780
	R: CGAATTGACATGAGTCACATTTACTTGTACAGCTCGTCCATGC	
*Klf4*	F: AATTACAGGCTAGCTATCAGGCCACCATGGCTGTCAGCGACGC	1499
	R: ACCTGCTTGCTTTAGCAGAGAGAAGTTTGTGGCGCCGCTGCCAAAGTGCCTCTTCATGTGTAAGG	
*Nanog*	F: CTGCTAAAGCAAGCAGGTGATGTTGAAGAAAACCCCGGGCCTATGAGTGTGGGCCCAGC	984
	R: CCCCGCATGTTAGAAGACTTCCCCTGCCCTCGCCGGAGCCCAAATCTTCAGGCTGTATGTTG	
*Lin28*	F: GTCTTCTAACATGCGGGGACGTGGAGGAAAATCCCGGCCCAATGGGCTCTGTGTCAAAC	702
	R: TCTCCAGCCAATTTCAAGAGAGCATAATTAGTACACTGGCCCGAGCCATTCTGGGCCTCTGGGAG	
*mCherry*	F: CTTGAAATTGGCTGGAGATGTTGAGAGCAACCCAGGTCCCATGGTGAGCAAGGGCGAG	771
	R: CGAATTGACATGAGTCACATTTACTTGTACAGCTCGTCCATGC	

F: Forward primer; R: Reverse primer.

**Table 3:** Pluripotency Gene Primer Information.

Gene	Primer (5′-3′)	Product Length (bp)
*endoOct4*	F: CAAGCAGTGACTACTCCCAACG	306
	R: ATCCCAAAGCCCTGGTACAA	
*endoSox2*	F: TTCGCCTGATTTTCCTCGC	518
	R: ATGAGCGTCTTGGTTTTCCG	
*endoNanog*	F: TTCCCAAACTACCCTCTCAAC	206
	R: CTCTTACTGGACTCATTACCCTTC	
*Sall4*	F: GCAGCGTTCCATCCACATTTAT	377
	R: CCTCTTTGCGTCCGTTCCTA	
*Rex1*	F: ACCTATTGTGGAAGAGGACCC	403
	R: ATCTCGGGGATTATAAACTCG	
*CDH1*	F: ACCCCCTGTCGGTGTTTTTAT	321
	R: GCTGGGATTGTGTAACCGAT	
*TERT*	F: GCGGAAGCCAAGTCTCGGAA	356
	R: GCGTGGTTCCCAAGCAGTTTC	
*STAT3*	F: TGAATGGAAACAACCAGTCGG	412
	R: CGCTGGACTACGAAGGCAC	
*GAPDH*	F: TGCTGGTGCTGAGTATGTGGT	613
	R: AGAAGAGTGAGTGTCGCTGTT	

F: Forward primer; R: Reverse primer.

## Data Availability

Data are contained within the article.
